# Insights of Outcome after Resection of Small Nonfunctioning Neuroendocrine Pancreatic Tumors

**DOI:** 10.1155/2021/6650386

**Published:** 2021-04-24

**Authors:** Estela Regina Ramos Figueira, Julia Fray Ribeiro, Thiago Costa Ribeiro, Ricardo Jureidini, Guilherme Naccache Namur, Thiago Nogueira Costa, Telesforo Bacchella, Ivan Cecconello

**Affiliations:** ^1^Hospital das Clinicas and Instituto do Cancer (ICESP) of University of São Paulo School of Medicine, Division of Digestive Surgery, Sao Paulo, Brazil; ^2^University of São Paulo School of Medicine, Scientific Research in Medicine FAPESP n° 2019/03584-0, Sao Paulo, Brazil

## Abstract

**Background:**

The incidence of small nonfunctioning neuroendocrine pancreatic tumors (NF-PNETs) has been increasing systematically in the last few decades. Surgical resection was once considered the treatment of choice but has been questioned in the direction of a more conservative approach for selected patients. Our aim was to analyze the outcome of surgical resection of small (≤3cm) NF-PNETs.

**Methods:**

We retrospectively evaluated 14 patients with sporadic NF-PNETs who underwent pancreatic resection. Data were collected from patients' medical records.

**Results:**

Of the 14 patients included, 35.71% were men, and the average age was 52.36 ± 20.36 years. Comorbidities were present in 92.86% of the cases. The incidence of postoperative complications was 42.86%, the 30-day mortality was zero, and the length of follow-up was 3.31 ± 3.0 years. The results of pathological evaluations revealed WHO grade I in 42.86% of cases, II in 21.43%, and neuroendocrine carcinoma in 35.71%. The median tumor size was 1.85cm (range, 0.5–3cm), and 2 cases had synchronous metastasis. The median TNM stage was IIa (range, I–IV). The disease-free and patient survival rates were 87.5% and 100% at 3 years and 43.75% and 75% at 10 years, respectively. The tumor pathological grade was significantly higher in head tumors than body-tail tumors, but there were no differences with respect to tumor size and TNM staging.

**Conclusion:**

A surgical approach to treat small sporadic NF-PNETs is safe with low mortality and high patient survival. Based on these data, small pancreatic head tumors can be more aggressive, suggesting that surgical resection is still the best option to treat small nonfunctioning PNETS. Thus, conservative treatment should be indicated very cautiously for only cases with absolute contraindications for surgery.

## 1. Introduction

Pancreatic neuroendocrine tumors (PNETs) are rare tumors with indolent behavior, representing 1 to 2% of all pancreatic cancers [1]. Most PNETs are sporadic and not related to inherited syndromes [2]. The frequency of these lesions continues to rise, which is probably related to the great improvement in imaging techniques [3]. Currently, the incidence of nonfunctioning PNETs (NF-PNETs) corresponds to 60-90% of all PNETs, with generally poorer prognosis than functional tumors [4].

The diagnosis of incidental nonfunctioning tumors represents more than 50% of all NF-PNETs. In the United States, the SEER registry has shown an increase in the incidence of small tumors by 710.4% over 22 years [5]. Many of these asymptomatic tumors are diagnosed in elderly patients with comorbidities. In this context, surgical resection is still the gold standard of treatment for NF-PNETs, which is considered the only curative option in most cases and has a positive impact on survival. Nevertheless, a controversy has arisen regarding the management of small NF-PNETs, at it has been proposed that small tumors may be observed in some cases [6]. Furthermore, in their 2012 and 2016 guidelines, the European Neuroendocrine Tumor Society (ENETS) has suggested a nonoperative approach to select benign small tumors ≤ 2 cm [7, 8]. Thus, the present study is aimed at analyzing the outcomes of surgical resection of small (≤3 cm) NF-PNETs.

## 2. Patients and Methods

Data were retrospectively reviewed from electronic medical records (HCMED and TAZY). The study included 14 patients with small NF-PNETs who underwent pancreatic resection with curative intention at Hospital das Clinicas da Faculdade de Medicina da Universidade de Sao Paulo (HCFMUSP) between August 1999 and October 2016. During this period of time, all patients were referred for surgical treatment except for those who had high risk according to the preoperative evaluation. Permission was first obtained from our institutional review board, and the study was conducted in accordance with the Declaration of Helsinki (1964).

Before the indication of surgical treatment, a multidisciplinary team including surgeons, oncologists, and radiologists evaluated all patients. Only patients with sporadic and asymptomatic NF − PNETs ≤ 3 cm were included in the study. The patient data collected included sex, age, weight, body mass index (BMI), Karnofsky performance status (KPS), American Society of Anesthesiologists (ASA) physical status, comorbidities, type of surgical resection, postoperative complications according to the Clavien-Dindo classification [9], postoperative pancreatic fistula, length of stay, follow-up, patient survival, and disease-free survival. Tumor data included pancreatic location, histologic grade according to the World Health Organization 2017 (WHO) classification [10], lymph node metastasis, synchronous liver metastasis, tumor size, TNM classification according to ENETS/AJCC [11, 12], and tumor recurrence.

### 2.1. Statistical Analysis

Values were expressed as the means and standard deviations, medians with the minimum and maximum, or percentages as appropriate. Medians were compared using the Mann-Whitney test. The Kaplan-Meier method was used to estimate the actuarial survival rates.

## 3. Results

The study included 14 patients, which comprised 35.71% men and had a mean age at surgery of 52.36 ± 20.36 years. The mean weight was 68.5kg, the mean BMI was 27.42 ± 4.31, and 14.28% were obese. The preoperative risk assessment showed a median KPS of 90% and ASA status of 2, with 92.86% of patients presenting comorbidities (Table 1).

The main comorbidities included high blood pressure in 6 cases (42.86%), diabetes mellitus in 4 cases (28.57%), cigarette smoking, hypothyroidism, and obesity in 2 cases each (14.29%), and coronary artery disease in 1 case (7.14%). The surgical procedures included 6 (42.86%) pancreatoduodenectomies, 3 (21.43%) distal pancreatectomies, 3 (21.43%) central pancreatectomies, and 2 (14.28%) enucleations performed in the neck and tail. Tumors were located in the head (6/14), neck (1/14), body (5/14), and tail (2/14) of the pancreas.

According to the Clavien-Dindo classification, 42.86% of patients had postoperative complications, with a mean of 0.93 ± 1.33 complications per patient. However, there were no severe complications, and only 14.28% of patients developed a postoperative pancreatic fistula. The mean length of hospital stay was 11.57 ± 9.82 days, there was no 30-day mortality, and the mean follow-up was 3.31 ± 3.0 years. Only 2 patients had tumor recurrence during follow-up (Table 2).

**Figure 1:**
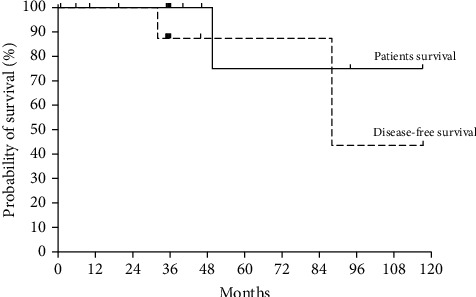
Postoperative patient survival and disease-free survival.

The pathological evaluation revealed WHO grade I in 42.86% of PNETs, grade II in 21.43%, and neuroendocrine carcinoma in 35.71%. The median tumor size was 1.85cm (range, 0.5–3cm). Two cases presented metastasis at surgery: one had a 1.6cm grade II tumor with lymph node and liver metastasis, and the other one had a 2.5cm grade II tumor with liver metastasis. The median T and TNM stages were I (range, I–II) and IIa (range, I–IV), respectively (Table 3). The disease-free and patient survival rates were 87.5% and 100% at 3 years and 43.75% and 75% at 10 years, respectively (Figure 1). An association between patient survival and tumor recurrence was not established.

Table 4 shows the tumor staging according to the pancreatic location of the tumor. The pathological WHO grade evaluation showed a significantly increased grade of head-neck tumors compared to body-tail tumors. However, there were no differences in tumor size and TNM-ENETS staging between groups.

## 4. Discussion

The best management of small sporadic nonfunctioning PNETs is controversial and still under debate. Although surgical resection is the standard recommended treatment with curative potential, there is a growing initiative for conservative management of small incidental NF-PNETs [13]. This is based on the better prognosis related to tumors smaller than 2 to 3 cm [14, 15]. Moreover, the ENETS 2016 guidelines still endorse surgical treatment for all resectable PNETs, and there is a trend toward conservative approaches to benign asymptomatic NF-PNETs that are ≤2 cm and located in the pancreatic head, with pancreatoduodenectomy being reserved for select cases [8].

A Canadian expert group recommends that NF-PNETs up to 2 cm in size with demonstrated low Ki67, tumor grades 1-2, and no evidence of malignancy can be selected for surveillance [16]. On the other hand, some have demonstrated more aggressive behavior in small tumors even with grades 1-2. Kuo and Salem [5] observed lymph node metastasis in 25% of 16 tumors with size of 0.1-0.5 cm and distant metastasis in 9.1% of 263 NF − PNETs ≤ 2 cm. Liu et al. [17] analyzed 1,226 PNETs ≤ 2 cm, including 224 (18.3%) cases with functioning tumors. They observed that 19.2% and 11.2% of the cases had distant and lymph node metastasis, respectively. Moreover, in this study, 71% of the resected pancreatic head tumors were neuroendocrine adenocarcinomas.

PNETs≤2 cm are increasingly diagnosed due to greater access to more accurate imaging tests, which are requested for other reasons but end up diagnosing these small tumors [18]. Current guidelines recommend surgical resection for this type of tumor, but the option for conservative treatment has gained strength based on the fact that small tumors have a better prognosis compared to larger tumors, fewer metastases, less malignant behavior, and no need for immediate surgery [13, 15, 19, 20]. Nonetheless, the main problem with this approach is the unpredictability of these highly heterogeneous small pancreatic tumors' behaviors, and determining their natural history with accuracy has been one of the biggest challenges [18]. In addition, the risk of malignancy of these tumors is not negligible, so surgical treatment would be associated with longer survival [19, 20].

In the present study, we selected only 14 patients diagnosed with small nonfunctioning PNETs with resectable tumors ranging from 0.5 to 3.0 cm in size. The mean follow-up was about three years, allowing for a reasonable period of observation. Although operative resection of pancreatic tumors can be related to morbimortality, it can certainly reduce tumor recurrence. All patients survived the surgical procedure and still presented 100% survival after 3 years. On the other hand, Sadot et al. [14] suggested a tumor size cutoff of 3.0 cm, while others suggested a cutoff of 2.0 cm in patients that could benefit from a more conservative approach [5, 6].

A message to point out in these cases subjected to expectant conduct is related to the necessity of a strict monitoring of patients through imaging examination every 3 months during the first year of follow-up, followed by every 6 months during the next 3 years. Furthermore, upon any signs of tumor growth or malignancy, the treatment must be switched immediately to surgical resection [7, 21]. However, small tumors can be malignant at presentation, as shown in this study, suggesting that surgical resection is still the best approach.

General preoperative health may have an impact on postoperative outcomes. The vast majority of patients included in this study (92.86%) were diagnosed with one or more comorbidities before surgery, which was to be expected since the age at initial diagnosis of this type of tumor ranges from 60 to 80 years [18]. This can be related to the amount of postoperative complications observed in 42.86% of patients, and among the possible complications, those that stand out included pancreatic fistulas, infections, hemorrhages, weight loss, and diabetes.

Among patients with complications, only 14.28% developed pancreatic fistulas, which is related to increased morbidity. Rosenberg et al. [22] observed a morbidity rate of 35% associated with patients undergoing surgery, with 25% showing pancreatic fistulas. However, Gaujoux et al. [18] obtained a total morbidity rate of 62% among operated patients, and the constancy of a higher rate of pancreatic fistula was maintained, representing 80% of complications.

Up to now, few prognostic markers have been used in clinical practice. In addition to tumor size, lymph node invasion, and distant metastasis in the TNM staging system, only the Ki67 proliferation index and mitotic rate have been used to grade PNETs and determine the prognosis [2]. An interesting point in choosing an expectant approach is that histological diagnosis of the tumor is not always possible, and some types of tumors are classically associated with a worse prognosis, such as neuroendocrine carcinomas. Bettini et al. [15] obtained a total of 90 patients with tumors up to 2 cm, and of these, 24.4% were of unknown histology. Of the 14 cases analyzed in this study, there were 5 cases of neuroendocrine carcinoma, representing 35.71% of the cases.

The patients were followed for an average of 3.31 years, and there were 2 (14.29%) cases of tumor recurrence during this period. Rosenberg et al. [22] observed a 10% rate of metastasis in a group undergoing surgery without significant differences in the number of metastases between that group and another group that received observational treatment. Among a total of 52 patients who underwent surgical resection, Kurita et al. [23] observed that three had lymph node metastases, one experienced recurrence after surgery, and two died of other diseases, but no death was related to the PNET.

In our research, the patient survival rate was 100% at 3 years and 75% at 10 years, and the deaths were unrelated to the tumor. Bettini et al. [15] obtained a survival rate of 97% in 5 years in cases of tumors smaller than or equal to 2 cm, and in those over 4 cm, the survival rate in 5 years was 92%. In general, no difference in overall mortality is observed in comparisons between groups undergoing surgery and groups with conservative management [22, 23].

We believe that the risks and benefits of operating on such tumors must be weighed, and the prognostic factors for each tumor should be better established. Thus, it would be possible to make better informed decisions regarding management, but there are no well-defined prognostic factors for this purpose yet [24]. Some centers use specific measures for this decision, such as tumor size (cutoff 2 cm), tumor grade, staging, and the patient's desire [24]. Others say that patients with comorbidities should receive conservative treatment [25]. The cutoff size for a tumor to be considered small and warrant conservative treatment is about 2 cm in various studies [16, 18, 19, 22, 26, 27]. However, Liu et al. [28] indicate that only children with tumors under 1 cm would undergo conservative treatment safely, whereas resection of tumors between 1 and 2 cm would beneficial. In contrast, Haynes et al. [29] found that no cutoff value is safe enough to avoid tumor progression.

This study had some obvious limitations, which were largely related to the very small number of cases evaluated. As a result, it is impossible to project the results to a larger scale. Moreover, there was no conservative treatment group for comparison of the results. This is related to one bias of our reference service, which transfers patients only referred to surgical treatment. Finally, the advanced age of most patients makes it difficult to discern the real causes of complications and deaths and whether they are due to the tumor alone or if there is an influence of comorbidities and basic conditions of patients.

## 5. Conclusion

Small PNETs have considerable risk of malignancy at presentation. In addition, surgical treatment is a safe approach for patients with PNETs. On the other hand, more research is needed to better elucidate cases that would benefit from an expectant approach and those who require immediate surgical resection of the tumor. We reinforce that a prospective randomized double-blind clinical trial would be necessary for these recurrent doubts to be resolved.

## Figures and Tables

**Table 1:** Demographic data.

Variables	Results
Male, %	35.71 (5/14)
Age, mean years with SD	52.36 ± 20.36
Weight, mean kg with SD	68.52 ± 12.84
BMI, mean kg/m^2^ with SD	27.42 ± 4.31
BMI ≥ 30%	14.28 (2/14)
KPS, median % (min-max)	90 (80–100)
ASA, median (min-max)	2 (1–3)
Comorbidities, %	92.86 (13/14)

BMI: body mass index; KPS: Karnofsky performance status; ASA: American Society of Anesthesiologists physical status.

**Table 2:** Postoperative complications and follow-up.

Variables	Results
Patients with any complication, %	42.86 (6/14)
Incidence of complications, mean/patient with SD	0.93 ± 1.33
Severe complication IIIb to V, %	0 (0/14)
Pancreatic fistula, %	14.28 (2/14)
Length of stay, mean days with SD	11.57 ± 9.82
Follow-up, mean years with SD	3.31 ± 3.0
30-day mortality, %	0 (0/14)
Tumor recurrence, %	14.28 (2/14)

**Table 3:** Tumor characteristics.

Variables	Results
Histologic grade	
Grade I, %	42.86 (6/14)
Grade II, %	21.43 (3/14)
Grade III, %	0 (0/14)
Neuroendocrine carcinoma, %	35.71 (5/14)
Lymph node metastases, %	7.14 (1/14)
Synchronous liver metastases, %	14.28 (2/14)
Largest tumor, median size in cm (min-max)	1.85 (0.5–3.0)
TNM stage, median (min-max)	IIa (I–3A)
T (tumor) stage, median (min-max)	I (I–II)

**Table 4:** Tumor staging according to pancreatic location of the tumor.

	Head-neck *n* = 7	Body-tail *n* = 7	*p* value
Pathologic WHO grade	III (I–IV)	I (I–II)	0.0152
Tumor size, cm	2.5 (0.5–3.0)	2.2 (0.5–2.7)	0.4744
ENETS staging system	IIIb (I–IV)	I (I–2a)	0.1515

Histologic grade IV means neuroendocrine carcinoma. All values are represented as median (min-max).

## Data Availability

The data used to support the findings are available on request.
